# Prospective, single-centre evaluation of the safety and efficacy of percutaneous coronary interventions following a decision tree proposing a no-stent strategy in stable patients with coronary artery disease (SCRAP study)

**DOI:** 10.1007/s00392-022-02054-7

**Published:** 2022-07-01

**Authors:** Ludovic Meunier, Matthieu Godin, Géraud Souteyrand, Benoît Mottin, Yann Valy, Vincent Lordet, Christian Benoit, Ronan Bakdi, Virginie Laurençon, Philippe Genereux, Matthias Waliszewski, Caroline Allix-Béguec

**Affiliations:** 1 Cardiology Department, Centre Hospitalier La Rochelle, La Rochelle, France; 2 Cardiology Department, Clinique St-Hilaire, Rouen, France; 3 Département de Cardiologie, CHU Clermont-Ferrand, ISIT, CaVITI, CNRS (UMR-6284), Université d′Auvergne, Clermont-Ferrand, France; 4 Clinical Trials Unit, Centre Hospitalier La Rochelle, La Rochelle, France; 5 Clinical Trials Center, Cardiovascular Research Foundation, New York, NY USA; 6 Morristown Medical Center, Gagnon Cardiovascular Institute, Morristown, NJ USA; 7 Medical Scientific Affairs, B.Braun Melsungen AG, Berlin, Germany; 8 Department of Internal Medicine and Cardiology, Charité - Universitätsmedizin Berlin, Campus Virchow, Berlin, Germany

**Keywords:** Coronary artery disease, Percutaneous coronary intervention, Angioplasty, Drug-eluting stent, Drug-coated balloon, Patient outcome assessment

## Abstract

**Aim:**

We evaluated a decision algorithm for percutaneous coronary interventions (PCI) based on a no-stent strategy, corresponding to a combination of scoring balloon angioplasty (SCBA) and drug-coated balloon (DCB), as a first line approach. Stents were used only in unstable patients, or in case of mandatory bailout stenting (BO-stent).

**Methods:**

From April 2019 to March 2020, 984 consecutive patients, including 1922 lesions, underwent PCI. The 12-month primary end-point was a composite of major adverse cardiac events (MACE) defined as all-cause death, nonfatal myocardial infarction, nonfatal stroke, and target lesion revascularization. Patients were classified into conventional or no-stent strategy groups according to the PCI strategy. In the no-stent strategy group, they were further classified into BO-stent or DCB-only groups. Their metal index was calculated by stent length divided by the total lesion length.

**Results:**

The no-stent strategy was applied in 85% of the patients, and it was successful for 65% of them. MACE occurred in 7.1% of the study population, including 4.2% of all-cause death. Target lesion revascularization was required in 1.4%, 3.6%, and 1.5% of patients in the conventional DES, BO-stent, and DCB-only groups, respectively. MACE occurred more often in the elderly and in those treated with at least one stent (metal index greater than 0).

**Conclusions:**

The no-stent strategy, i.e., revascularization of coronary lesions by SCBA followed by DCB and with DES bailout stenting, was effective and safe at 1 year. This PCI approach was applicable on a daily practice in our cath lab.

**Trial registration:**

This study was registered with clinicaltrials.gov (NCT03893396, first posted on March 28, 2019).

**Graphical abstract:**

Feasibility, safety and efficacy of percutaneous coronary interventions following a decision tree proposing a no-stent strategy in stable patients with coronary artery disease. DES: drug eluting stent; SCBA: scoring balloon angioplasty; BO-stent: at least one stent; DCB: drug coated balloon; BMS: bare metal stent; Bailout (dash lines); MACE: major adverse cardiac event

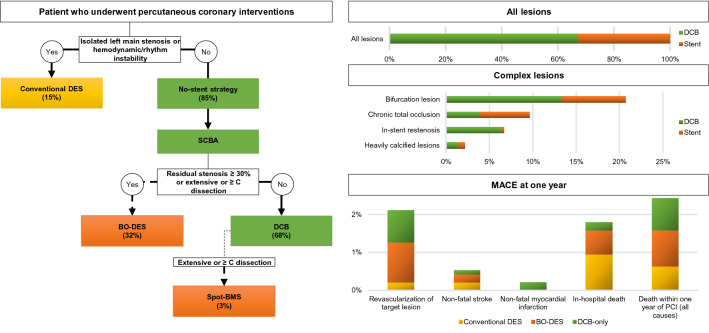

## Introduction

In 1977, Grüntzig started the non-surgical treatment of coronary artery disease, with balloon angioplasty [1]. Despite its novelty, this approach had three major limitations: the recoil reducing the acute luminal gain, the binary restenosis rate of 30–50% at 12 months, and the risk of acute occlusions in 2–10% of all procedures.

To overcome these complications, coronary angioplasty and stent insertion began in the 1990s with bare-metal stents (BMS). To reduce in-stent restenosis, first-generation and second-generation drug-eluting stents (DES) have been used since the 2000s. Stenting is currently the first-line treatment for all types of lesions in patients. The disadvantage of DES is that it causes stent thrombosis, which increases the ischemic risk in patients [2]. Dual antiplatelet therapy is prescribed, but this in turn is responsible for an increased risk of bleeding. Bioresorbable coronary stents were developed in order to eliminate permanent scaffolding and to restore endothelial function, but the reported rate of 2.1% stent thrombosis at 6 months was too high.

Drug-coated balloon (DCB) technology led to a 2014 ESC 1A recommendation for the treatment of in-stent restenosis [3]. Since DCBs have begun to be used successfully in de novo lesions, particularly in small vessels [4]. DCB angioplasty and the rational strategy to avoid stenting in primary angioplasties, i.e., the “No-Stent” Strategy”, have the merit of eliminating the major drawback of plain old balloon angioplasty (POBA) while leaving no foreign body implant [5].

We have implemented a decision algorithm for percutaneous coronary intervention (PCI) leading to conventional DES or a no-stent strategy with stenting as a safeguard in case of mandatory bailout (BO). In this single-centre prospective study, we aimed at evaluating the safety and efficacy of this decision algorithm using a 1-year composite clinical endpoint of major adverse cardiac events (MACE), consisting of all-cause death, non-fatal myocardial infarction, non-fatal stroke and target vessel revascularization.

## Methods

### Study design

Prospective, single-centre study of safety and efficacy of percutaneous coronary intervention.

### Setting

The interventional cardiology unit of a public hospital was staffed with five interventional cardiologists of different levels of experience (three seniors, one junior, and one part-time cardiologist) in coronary angioplasty and stenting.

### Participants

All consecutive patients in whom coronary angioplasty was performed, i.e., acute coronary syndrome non-ST myocardial infarction (NSTEMI) or ST myocardial infarction (STEMI), chronic coronary syndrome including angina, silent ischemia, ischemic heart disease, were eligible to be included in this study. The exclusion criteria were patients < 18 years of age, pregnancy, legally protected patients or deprived of liberty, and refusal to participate.

### Percutaneous coronary intervention

If the patient was hemodynamically or rhythmically unstable, conventional DES (C-DES) angioplasty was performed. Unstable patients were those in shock, with ventricular hyperexcitability (ventricular tachycardia, ventricular fibrillation), or at risk of instability if the no-stent strategy were used with prolonged inflation of DCB (multi vessels and left ventricular ejection fraction ≤ 30%, isolated left main coronary artery). The available DES were the Xience (Abbott Vascular Rungis, France), Synergy (Boston Scientific, Voisins-le-Bretonneux, France), and Onyx (Medtronic France SAS, Paris, France).

In patients eligible for a no-stent strategy, lesion preparation was done with scoring balloons (NSE Alpha, Nipro Europe, Michelen, Belgium) angioplasty (SCBA). In situations of persistent residual stenosis ≥ 30%, or the occurrence of a dissection at high risk of acute occlusion (≥ C [6], and regardless of vessel diameter), or a dissection at risk of secondary aneurysmal (≥ B, if it is extensive and occurs in a large-calibre vessel), bailout stenting with DES was performed (BO-DES). If the lesion preparation was successfully done, the second step of angioplasty was performed with DCB (SeQuent Please Neo, B.Braun Melsungen AG, Germany). In situations of significant dissection, spot bailout stenting of the dissection was performed with a bare metal stent (Optimax, Hexacath, Rueil-Malmaison, France) (spot-BMS).

The following situations may have arisen and required adaptations: 

- In case of a highly calcified sub occlusive lesion, rotational atherectomy (Rotablator, Boston Scientific) could be performed before SCBA.

- In case of bifurcation lesion of Medina type 011 or 111, the scoring was done first in the main branch and then in the side branch. After lesion preparation, DCB were first used in the side branch and then in the main branch. In the other types of Medina, bifurcation lesions were treated in the same way as de novo lesions.

- In acute thrombotic occlusion (STEMI) with a high thrombus burden, a Minimalist Immediate Mechanical Intervention (MIMI) attitude was advocated before the no-stent strategy, because inflation of the scoring balloon would present a risk of distal thrombus embolization, and a high thrombus burden would significantly reduce DCB drug delivery.

### Variables

The primary end-point was a composite of MACE within 12 months of the procedure. MACE were defined as all-cause death, nonfatal myocardial infarction, nonfatal stroke, and target lesion revascularization.

### Data sources/measurement

Clinical and procedural data were retrieved from CardioReport™ software (MediReport). Follow-up data were obtained by phone, consultation, or hospital admission. Angiographic variables were calculated as follows. The total length of lesions treated (TLL) is the total length of DES and DCB used per patient. The mean diameter is the sum of the diameter of each material multiplied by its length, and the whole divided by the TLL. The metal index is the total length treated with a DES (or in very scarce cases with a BMS in first line) divided by the TLL. The data underlying this article are available in Zenodo [7].

### Statistical analysis

Means ± standard deviations or median and interquartile range, counts and percentages were used to describe continuous and categorical variables, respectively. Normal distributions were verified and *T* test or Kruskal–Wallis or Mann–Whitney tests were used to compare continuous data of independent samples where appropriate. Chi-square or Fisher test of homogeneity was used for categorical variables. An alpha level of 0.05 was used for all statistical tests. Risk factors for MACE were first analysed by bivariate analysis, then a selection of predictors were added in a logistic regression model.

## Results

### Study population

From April 2019 to March 2020, 984 consecutive patients, totalizing 1922 lesions, underwent interventional revascularization performed during 1136 PCI. One patient declined to participate.

**Fig. 1:**
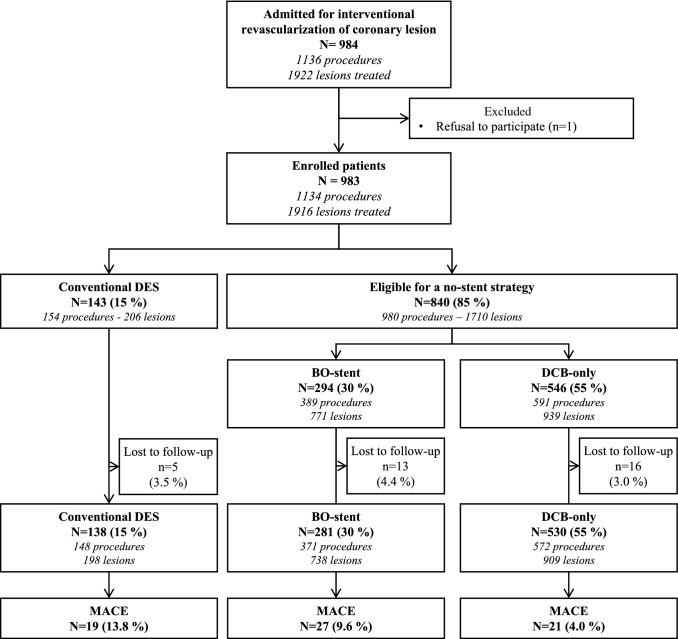
Flow diagram. DES: drug eluting stent; BO-stent: at least one stent; DCB: drug coated balloon; MACE: major adverse cardiac event

Fifteen percent of patients where unstable and required conventional DES, while 85% of the study population was eligible for a no-stent strategy (Fig. 1). Revascularization with DCB-only was performed in 546 patients. The remaining 294 patients required at least one stent and were classified in the BO-stent group. This group included 137 patients revascularized exclusively by BO-DES after scoring (residual stenosis ≥ 30%, or dissection at high risk of acute occlusion or late secondary aneurysmal), 132 patients who had hybrid revascularization with both BO-DES and DCB, and 25 patients who had spot-BMS after DCB.

### Patient characteristics

Three-quarters of the patients were male, and the mean age was 69 ± 12 years. Clinical characteristics and cardiology history were similar in patients in the conventional DES, BO-stent, or DCB-only groups, with the exception of family history of coronary artery disease, tobacco use, and clinical presentation (Table 1). One-third of stable patient had small-vessel disease.

**Table 1:** Characteristics of patients treated with a conventional or no-stent strategy

	Conventional DES		Eligible for a no-stent strategy				*p* value
			BO-stent		DCB-only		
	(*n* = 143)		(*n* = 294)		(*n* = 546)		
*Demographics*							
Age, year	68.4	(± 13.5)	69.0	(± 11.4)	68.4	(± 11.3)	0.914
Men, number	102	(71%)	222	(76%)	409	(75%)	0.618
Mean BMI, kg/m^2^	27.3	(± 4.5)	27.4	(± 4.4)	27.4	(± 5.0)	0.945
*Clinical characteristics*							
Family history of coronary artery disease	16	(11%)	47	(16%)	152	(28%)	** < 0.001**
Diabetes	38	(27%)	75	(26%)	125	(23%)	0.600
High blood pressure	67	(47%)	157	(53%)	262	(48%)	0.241
Hypercholesterolemia	67	(47%)	55	(50%)	268	(48%)	0.268
*Smoking status*							
No smoker	79	(55%)	145	(49%)	259	(48%)	**0.037**
Current smoker	34	(24%)	68	(23%)	101	(19%)	
Former smoker	30	(21%)	81	(28%)	180	(33%)	
*Cardiology history*							
Angioplasty	31	(22%)	58	(20%)	139	(26%)	0.128
Coronary artery bypass graft	9	(6%)	11	(4%)	25	(5%)	0.490
Myocardial infarction	23	(16%)	22	(7%)	41	(8%)	0.004
Stroke	4	(3%)	6	(2%)	13	(2%)	NA
Lower limb peripheral arterial disease	6	(4%)	19	(6%)	30	(6%)	0.624
*Presentation*							
STEMI	58	(41%)	54	(18%)	61	(11%)	** < 0.001**
NSTEMI	37	(26%)	87	(30%)	120	(22%)	
Chronic coronary syndrome	48	(34%)	153	(52%)	365	(67%)	
*Multivessel disease*							
Single vessel	114	(80%)	137	(47%)	396	(73%)	** < 0.001**
Multi vessels	29	(20%)	157	(53%)	150	(27%)	
*Lesions treated*							
Total length of lesion treated, mm	35.5	(± 23.9)	69.1	(± 45.1)	51.5	(± 33.5)	** < 0.001**
Mean diameter, mm	3.2	(± 0.4)	3.1	(± 0.4)	3.1	(± 0.4)	**0.028**
Small-vessel (< 3 mm)	35	(24%)	161	(29%)	114	(39%)	**0.003**
Metal index	1.0	(± 0.0)	0.6	(± 0.3)	0.0	(± 0.0)	** < 0.001**
*Device*							
Number of devices	1.4	(± 0.7)	2.7	(± 1.5)	1.7	(± 1.0)	** < 0.001**

Data are presented as means ± standard deviations or as counts and percentages

Values in bold correspond to *p* values lower than 0.05 considered as the threshold of statistical significance

DES: drug-eluting stent; BO-stent: at least one stent; DCB: drug coated balloon; BMI: body mass index; STEMI: acute ST-elevation myocardial infarction; NSTEMI: non-ST segment elevation myocardial infarction; NA: not applicable; MACE: major adverse cardiac event

### Procedures

**Fig. 2:**
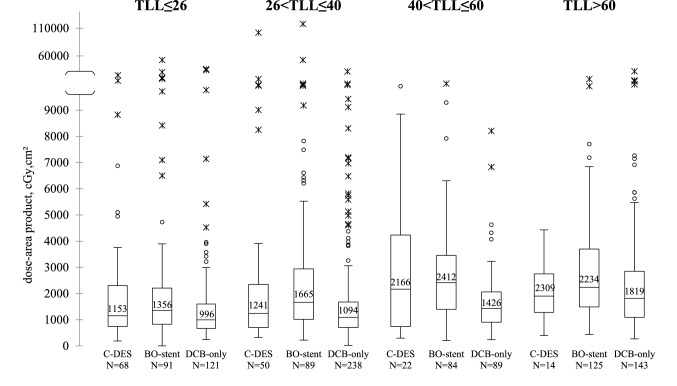
Dose-area product per category of total length of lesions treated and per device. In the box plots, the black centre lines denote the median value of dose-area product. The numbers above these lines are the median value. The boxes contain the 25th and 75th percentiles. Rounds and crosses above the whiskers' upper bounds may be considered as outliers. C-DES: conventional drug-eluting stent strategy; BO-stent: at least one stent; DCB: drug coated balloon; TLL: total length of lesions treated (mm)

DCB-only strategy had similar procedure times and contrast volumes as conventional DES strategy (Table 2). BO-stent procedures took longer and required more contrast medium. Fluoroscopy times were related to the total length of the treated lesions. Radiation dose were similar in conventional DES and DCB-only groups (Fig. 2). Rotational atherectomy was used in 42 procedures to treat lesions with a high calcium content. In two-thirds of the cases (*n* = 27, 64%), these lesions were successfully revascularised with a no-stent strategy.

**Table 2:** Characteristics of the procedures according to the strategy used

	Conventional DES		Eligible for a no-stent strategy				*p* value
			BO-stent		DCB-only		
	(*n* = 154)		(*n* = 389)		(*n* = 591)		
Fractional flow reserve	6	(4%)	19	(5%)	19	(3%)	0.416
Rotablator	3	(2%)	12	(3%)	27	(5%)	
Time length, min	31	[23–46]	39	[30–55]	31	[23–42]	< 0.001
Liquid contrast volume, ml	180	[150–213]	220	[160–280]	180	[150–220]	< 0.001
*Fluoroscopy time length, min*							
TLL ≤ 26	9	[6–14]	9	[6–12]	8	[6–10]	0.032
26 < TLL ≤ 40	8.13	[6–14]	9.47	[7–13]	8.17	[6–11]	0.192
40 < TLL ≤ 60	9.57	[6–13]	11.05	[8–16]	10	[8–14]	0.217
TLL > 60	7.68	[6–18]	12.44	[9–18]	11.82	[9–16]	0.443

Data are presented as counts and percentages or as median and interquartile range

DES: drug-eluting stent; BO-stent: at least one stent; DCB: drug coated balloon; TLL: total length of lesions treated (mm)

### Lesions treated

Of the 1916 lesions, 33% were treated with DES and 67% with DCB (Table 3). In stable patients, 420 lesions required BO-DES. For lesions treated with DCB, 28 (2.2%) required secondary bailout stenting (28 spot-BMS). In-stent restenosis, bifurcation lesions, and chronic total occlusion (CTO) accounted for 7%, 21%, and 10% of treated lesions, respectively. Most in-stent restenosis (96%) and 64% of bifurcation lesions were successfully treated with a no-stent strategy. For CTO, the no-stent strategy succeeded in only 40% of the lesions.

**Table 3:** Description of the total lesions treated and according to the device used

	Treated lesions		DES		DCB	
	(*n* = 1916)		(*n* = 626)		(*n* = 1290)	
Right coronary artery	542	(28%)	200	(32%)	342	(27%)
Left main coronary artery	88	(5%)	74	(12%)	14	(1%)
Left anterior descending artery	843	(44%)	238	(38%)	605	(47%)
Left circumflex artery	432	(23%)	109	(17%)	323	(25%)
Bypass	11	(1%)	5	(1%)	6	(0%)
In-stent restenosis	128	(7%)	5	(1%)	123	(10%)
Bifurcation lesion	397	(21%)	142	(23%)	255	(20%)
Chronic total occlusion	185	(10%)	111	(18%)	74	(6%)

Data are presented as counts and percentages

DES: drug eluting stent; DCB: drug coated balloon

### Concomitant drug treatment

Dual antiplatelet therapy (DAPT) was initiated with a prescription for at least 1 month. Ongoing treatment data were available for 868 patients (88%) at 1 year (Table 4). The majority of patients treated for chronic coronary syndrome with DCB-only (64%) were on single antiplatelet therapy (SAPT). In patients treated for acute coronary syndrome, more than half were still on DAPT.

**Table 4:** Drug treatment at 1 year

	Acute coronary syndrome						Chronic coronary syndrome					
	C-DES		BO-stent		DCB-only		C-DES		BO-stent		DCB-only	
	(*n* = 72)		(*n* = 118)		(*n* = 159)		(*n* = 42)		(*n* = 140)		(*n* = 337)	
DAPT	46	(64%)	68	(58%)	76	(48%)	19	(45%)	60	(43%)	57	(17%)
SAPT	19	(26%)	39	(33%)	60	(38%)	17	(40%)	60	(43%)	215	(64%)
DAPT + OAC	1	(1%)	1	(1%)	3	(2%)	0	(0%)	5	(4%)	5	(1%)
SAPT + OAC	6	(8%)	6	(5%)	11	(7%)	4	(10%)	8	(6%)	27	(8%)
OAC	0	(0%)	4	(3%)	9	(6%)	2	(5%)	7	(5%)	33	(10%)

Data are presented as counts and percentages

C-DES: conventional drug eluting stent; BO-stent: at least one stent; DCB: drug coated balloon; DAPT: dual antiplatelet therapy; SAPT: single antiplatelet therapy; OAC: oral anticoagulant

### Major adverse cardiac events

**Fig. 3:**
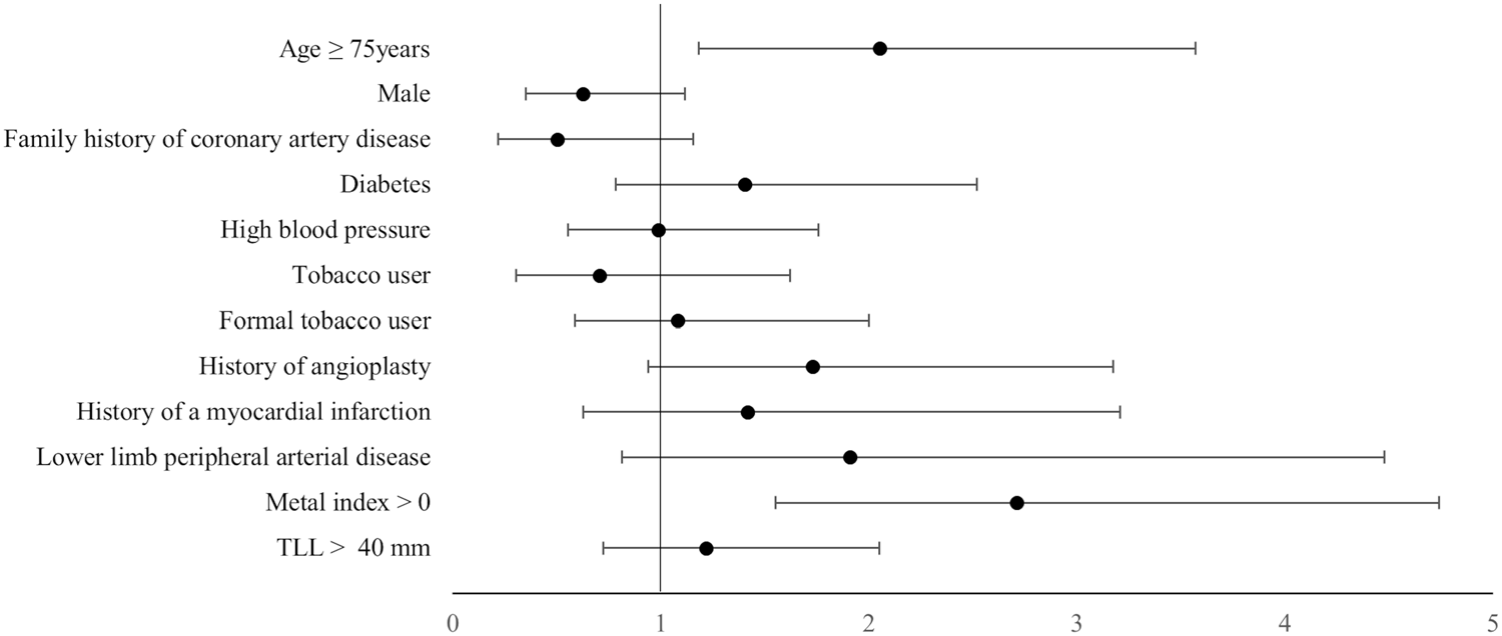
Risk factors of major adverse cardiac events (MACE) among the study population. TLL: total length of lesions treated (mm)

During the 1-year follow-up, MACE occurred in 7.1% of the study population (Table 5). Death from any cause affected 4.2% of the study population (10.9%, 5.3% and 1.9% in the conventional DES, BO-stent and DCB-only group, respectively). Seventeen patients died in hospital after angioplasty. Twelve patients in critical state deceased due to cardiopulmonary arrest and/or STEMI complicated by cardiogenic shock or multi-organ failure. Five deaths were due to iatrogenic causes, three in the conventional DES group (two probable stent thrombosis and one acute renal failure), and two in the DCB-only group (one left main coronary artery dissection and one haemorrhagic stroke). Target lesion revascularization was required in 20 patients (1.4%, 3.6% and 1.5% of patients in the conventional DES, BO-stent and DCB-only groups, respectively). Five patients had non-fatal stroke (two in the conventional DES group, two in the BO-stent group, and one in the DCB-only group). Two patients in the DCB-only group experienced non-fatal myocardial infarction. MACE occurred more often in elderly and in those treated with at least one stent (metal index greater than 0) (Fig. 3).

**Table 5:** Type of major adverse cardiac events in the study population and according to the strategy used

	Study population		Conventional DES		Eligible for a no-stent strategy			
					BO-stent		DCB-only	
	(*n* = 949)		(*n* = 138)		(*n* = 281)		(*n* = 530)	
Revascularization of target lesion	20	(2.1%)	2	(1.4%)	10	(3.6%)	8	(1.5%)
Non-fatal stroke	5	(0.5%)	2	(1.4%)	2	(0.7%)	1	(0.2%)
Non-fatal myocardial infarction	2	(0.2%)	0		0		2	(0.4%)
In-hospital death	17	(1.8%)	9	(6.5%)	6	(2.1%)	2	(0.4%)
Death within 1 year of PCI (all causes, in-hospital death excluded)	23	(2.4%)	6	(4.3%)	9	(3.2%)	8	(1.5%)
Total MACE	67	(7.1%)	19	(13.8%)	27	(9.6%)	21	(4.0%)

Data are presented as counts and percentages. DES: Drug Eluting Stent; BO-stent: at least one stent; DCB: Drug Coated Balloon; PCI: percutaneous coronary intervention; MACE: major adverse cardiac event

## Discussion

This study reports the 1-year clinical outcomes at a high-volume interventional cardiology centre following the intention to treat PCI patients without permanent implants. The 1-year MACE rate was 7.1%, mainly related to all-cause death (4.2%), with the target lesion revascularization rate remaining low at 2.1%, and the rate of non-fatal stroke and infarction being rare (0.5% and 0.2%, respectively).

Our decision tree mostly follows the recommendations of the third report of the international DCB consensus group [8]. The only difference concerns the preparation of the lesions with exclusively the scoring balloon in our study, whereas the recommendations leave the choice between compliant, semi-compliant, scoring, and cutting balloon. Comparison of our all-comer study of patients classified into three subgroups (conventional DES, BO-stent, and DCB-only) with other published studies should be taken with caution. On the one hand, either study populations are selected samples based on clinical or angiographic data, or they include all-comers but focus on only one type of device. On the other hand, the subgroup consisting of BO-stent is typically not well documented. The current knowledge with a high level of evidence comparing DCB with DES is based on three randomized controlled trials. In a study of de novo lesions in small coronary vessels, the 1-year MACE rate was 7.5% in both the DCB and the DES groups, demonstrating the non-inferiority of DCB angioplasty [4]. In patients at high risk of bleeding, the 9-month MACE rate after DCB angioplasty was 1% compared with 14% in the BMS control group [9]. In NSTEMI patients, a MACE rate of 6.7% was reported after DCB angioplasty, compared with 14.2% in a BO-stent group of patients treated with either BMS or DES [10]. With the exception of the BASKET–SMALL 2 study [4], data variability and study designs, particularly for the study with BMS in the control group, hinder meaningful comparisons with our study results. All-comers observational studies and registries are available but focused on one device. In our DES group, the 1-year MACE rate is higher than the 5.8% MACE rate reported in a study, where patients were treated with DES exclusively [11]. This difference may be explained by the in-hospital mortality rate of 6.5% reported in our study. This is probably due to our revascularization algorithm, in which unstable patients or those at risk of being unstable, i.e., the most severe, were directly treated with DES. In these patients, prolonged inflation of the DCB would have been very poorly tolerated due to transmural ischemia. Our MACE rate of 4.0% in the DCB-only group is consistent with the reported 9-month MACE rate of 6.8% in the Rosenberg registry [12] and the 1-year MACE rate of 3.9% reported by Pan et al. [13].

Among patients discharged alive, the 1-year mortality rates in the conventional DES, BO-stent and DCB-only groups were 4.3%, 3.2%, and 1.5%, respectively. This is higher than the 1.5% all-cause deaths reported by Maupas et al*.* for patient treated with DES [11] but consistent with other registry data and depending of STEMI rate in the different population. For patients treated with DCB-only, these data are consistent with the reported cardiac death rate of 0.86–1.3% [12, 13]. In a meta-analysis of 26 randomized clinical trials comparing 4590 patients treated with DES or DCB, no significant difference in all-cause mortality was observed at 6–12 months, with a rate about 2% [14]. Scheller et al. also concluded that there was a trend toward lower mortality in patients treated with DCB compared with the DES population after 3 years of follow-up. Target lesion revascularization rates were low in the conventional DES and DCB-only groups (1.4% and 1.5%, respectively), which is similar to the 1.7% rate reported for DES [11], and the 1.9–3.2% rates reported for DCB [12, 13, 15]. Overall, our results are similar with currently available data from randomized control trials comparing DCB to DES in patients with small-vessel coronary artery diseases, which support comparable clinical outcomes between the two strategies [8].

Ideally, we would have preferred to study lesion by lesion rather than create a BO-stent group but the clinical analyses would have been impossible. The BO-stent group is typically excluded from studies and may represent one-third of the patients [13]. It includes patients for whom primary and secondary bailouts were necessary for at least one of their lesions, and patients affected by multivessel coronary artery disease. Multiple lesions imply a greater length of lesions treated, and a greater number of devices used. One hypothesis is that by multiplying the number of devices, the lesions of the media, linked to the multiplication of overlaps, are accentuated, which increases the risk of restenosis [16]. To treat a long lesion, a single long device might be more appropriate.

In the no-stent strategy group, the procedures included a first step of lesion preparation with balloon scoring. As shown in the PASSWORD study, the systematic use of a scoring balloon seems to guarantee the success of the intervention, whose good outcome is maintained over time [15]. Type A and B dissections are generally associated with acceptable angiographic outcomes and vessel healing [17], and a primary bailout stenting approach is not mandatory. However, dissections of type B in large calibre vessels (≥ 4 mm) in hypertensive patients are associated with high vessel wall stress according to Laplace’s law (wall stress is proportional to arterial pressure and vessel diameter and inversely proportional to the wall thickness), and patients are at risk for coronary aneurysm formation [18]. For this reason, we performed primary bailout stenting in these dissection subsets.

With the exception of unstable patients or those at risk of being unstable, the no-stent strategy was applied to all lesions, including complex lesions (bifurcation, calcification, CTO, in-stent restenosis). Classical treatments of bifurcation lesions are based on the use of one or two stent with several revascularization techniques [19]. In our study, two-thirds of bifurcation lesions were successfully treated with a no-stent strategy. Rotational atherectomy is classically used to treat lesions with a high calcium content [20]. Our results are consistent with those of Rissanen et al*.*, [21], which shows the feasibility of treating these lesions with a no-stent strategy. CTO may be processed with a no-stent strategy [22], but in our study, 60% of these lesions required BO-DES. As recommended in the 2014 ESC/EACTS guidelines, DCB were used to treat in-stent restenosis [3].

The metal index used in this study allowed classification of patients treated only with DES (metal index of 1), those treated with DCB-only (metal index of 0), and patients treated with both BO-DES and DCB (BO-stent) who are characterized by an intermediate metal index. From our results, the metal index could be used as a predictor of two events, each with a different time horizon. The first event was early, causal, and patient-related. In-hospital mortality was higher in patients with the highest metal index, as the most severe patients were treated with DES. The second event was later, consecutive, and concerns the ischemic consequences of a potential stent thrombosis. Moving towards the idea of a metal index and its long-term effect, Ejiri et al*.* showed an association between total stent length and increased risk of annual MACE [23].

For equivalent procedure duration and total length of lesions treated, the dose-area product was low in the no-stent strategy group. Compared with procedures with DES, the radiation dose required during full stenting optimization, including the complex procedures used to treat bifurcations, such as POT, rePOT, kissing, DK crush, and stent boost, was not necessary once DCB angioplasty was done.

At 1 year, the majority of patients treated for chronic coronary syndrome with DCB-only were on SAPT without excess risk of MACE. The no-stent strategy may become a preferential method of angioplasty depending on the patient’s clinical situation and lesion type. It would primarily benefit high-risk ischemic patients, such as young patients by avoiding the cumulative annual risk of very late stent thrombosis, and high-risk bleeding patients, such as elderly or patients on oral anticoagulant for easier management of bleeding risk using a short DAPT or a SAPT.

### Limitations and perspectives

MACE at 1 year does not take into consideration the ischemic risk related to very late stent thrombosis, whose cumulative rate is 0.2–1% per year [24, 25]. The study will be extended to allow for a 3-year follow-up and to assess the longer term ischemic risk and prognostic ability of the metal index, which will allow to compare the results with those of BASKET–SMALL 2 at 3 years [26]. The study design focused on the ischemic risk without studying the haemorrhagic risk caused by major bleedings, and could not characterize the ischemic/haemorrhagic balance of NACE (Net Adverse Clinical Events), as in the TICO [27] and TWILIGHT [28] studies. This prospective, single-centre study has a lower level of evidence than multicentre randomized controlled studies, but its real-life design including almost all consecutive cases limits selection.

We believe that improving the efficacy and safety of the no-stent strategy could be achieved in two key steps. (1) The observed BO-DES rate of around 24% could be improved. For example, intravascular lithotripsy (shock wave) may help in the treatment of severely calcified lesions in coronary vessels and limit the rate of dissection [29]. (2) Although the target lesion revascularization rate is already low, recently available Sirolimus-coated balloons may improve specific treatments, such as in-stent restenosis, or limit the subsequent risk of excessive late luminal enlargement [30].

## Conclusions

For stable patients, the no-stent strategy, i.e., revascularization of coronary lesions by scoring balloons followed by DCB, with DES stenting in bailout, is an effective strategy with a low 1-year MACE rate. It has been applicable to the majority of patients’ lesions, including complex lesions such as bifurcations and heavily calcified lesions. This strategy is not time-consuming and is associated with a low radiation dose.
