# Correction: Prospective, single-centre evaluation of the safety and efficacy of percutaneous coronary interventions following a decision tree proposing a no-stent strategy in stable patients with coronary artery disease (SCRAP study)

**DOI:** 10.1007/s00392-022-02073-4

**Published:** 2022-08-04

**Authors:** Ludovic Meunier, Matthieu Godin, Géraud Souteyrand, Benoît Mottin, Yann Valy, Vincent Lordet, Christian Benoit, Ronan Bakdi, Virginie Laurençon, Philippe Genereux, Matthias Waliszewski, Caroline Allix-Béguec

**Affiliations:** 1 Cardiology Department, Centre Hospitalier La Rochelle, La Rochelle, France; 2 Cardiology Department, Clinique St-Hilaire, Rouen, France; 3 Département de Cardiologie, CHU Clermont-Ferrand, ISIT, CaVITI, CNRS (UMR-6284), Université d′Auvergne, Clermont-Ferrand, France; 4 Clinical Trials Unit, Centre Hospitalier La Rochelle, La Rochelle, France; 5 Clinical Trials Center, Cardiovascular Research Foundation, New York, NY USA; 6 Morristown Medical Center, Gagnon Cardiovascular Institute, Morristown, NJ USA; 7 Medical Scientific Affairs, B.Braun Melsungen AG, Berlin, Germany; 8 Department of Internal Medicine and Cardiology, Charité - Universitätsmedizin Berlin, Campus Virchow, Berlin, Germany

## Correction: Clinical Research in Cardiology 10.1007/s00392-022-02054-7

The article “Prospective, single-centre evaluation of the safety and efficacy of percutaneous coronary interventions following a decision tree proposing a no-stent strategy in stable patients with coronary artery disease (SCRAP study)”, written by Ludovic Meunier, Matthieu Godin, Géraud Souteyrand, Benoît Mottin, Yann Valy, Vincent Lordet, Christian Benoit, Ronan Bakdi, Virginie Laurençon, Philippe Genereux, Matthias Waliszewski and Caroline Allix-Béguec, was originally published electronically on the publisher’s internet portal on 1 July without open access. With the author(s)’ decision to opt for Open Choice the copyright of the article changed on 11 July to © The Authors 2022 and the article is forthwith distributed under a Creative Commons Attribution 4.0 International License, which permits use, sharing, adaptation, distribution and reproduction in any medium or format, as long as you give appropriate credit to the original author(s) and the source, provide a link to the Creative Commons licence, and indicate if changes were made.

The images or other third party material in this article are included in the article’s Creative Commons licence, unless indicated otherwise in a credit line to the material. If material is not included in the article’s Creative Commons licence and your intended use is not permitted by statutory regulation or exceeds the permitted use, you will need to obtain permission directly from the copyright holder.

To view a copy of this licence, visit http://creativecommons.org/licenses/by/4.0/.

The original article has been corrected.

